# The force-temperature relationship in healthy and dystrophic mouse diaphragm; implications for translational study design

**DOI:** 10.3389/fphys.2012.00422

**Published:** 2012-11-07

**Authors:** Jason D. Murray, Benjamin D. Canan, Christopher D. Martin, Jenna E. Stangland, Neha Rastogi, Jill A. Rafael-Fortney, Paul M. L. Janssen

**Affiliations:** ^1^Department of Physiology and Cell Biology, The Ohio State University, Columbus, OH, USA; ^2^Department of Molecular and Cellular Biochemistry, The Ohio State University, Columbus, OH, USA

**Keywords:** muscle strips, physiology, contraction, *mdx*, Duchenne muscular dystrophy

## Abstract

In the field of muscular dystrophy, striated muscle function is often assessed *in vitro* in dystrophin-deficient *mdx* mice in order to test the impact of a potential treatment strategy. Although many past studies have assessed diaphragm contractile function at or near room temperature, the diaphragm performs *in vivo* at 37°C. To improve translation of bench-top results to possible clinical application, we studied temperature-dependence of contractile performance in wild-type (C57BL/10) and *mdx* muscle strips at temperatures from 25°C to 37°C. Maximal tetanic force in wild-type muscles was higher at 37°C (198 ± 11 vs. 155 ± 9 mN/mm^2^ at 25°C), while the difference between wild-type and *mdx* was extremely similar: wild-type muscles produced 45.9% and 45.1% more force at 25°C and 37°C respectively. At 37°C twitch contraction kinetics and 50% rise time to tetanic plateau were slower in *mdx* diaphragm. A fatigue/injury protocol indicated 2-fold fatigue/contraction-induced force deficit in *mdx* muscles. We conclude that assessment of diaphragm muscle strips can be reliably and reproducibly performed at 37°C.

## Introduction

Duchenne muscular dystrophy (DMD) is an X-linked disorder that affects males and is characterized by muscle degeneration and weakness. This disease results in death by the third decade of life due to cardiac and respiratory complications (Emery, 2002). In order to assess therapeutic benefit of genetic or pharmacological interventions, the *mdx* mouse is a widely employed model of DMD (Bulfield et al., 1984; Stedman et al., 1991). *In vitro* contractile parameters of isolated muscle strips dissected from the mouse diaphragm are commonly reported throughout muscle physiology literature (Petrof et al., 1993a; Stevens and Faulkner, 2000; Brooks et al., 2002; Blaauw et al., 2009), and has been widely used for functional evaluation of animal models of health and disease (Stedman et al., 1991; Gregorevic et al., 2002; Acharyya et al., 2007; Martin et al., 2009), determination of cross-bridge kinetics (Coirault et al., 1999a), and as a quantifiable outcome evaluator when considering therapeutics (Gosselin and Williams, 2006; Peterson et al., 2011; Rafael-Fortney et al., 2011).

There exists some degree of irregularity when comparing reported values relating to contractile function of isolated diaphragm muscle, especially among observed tetanic force measurements in healthy control mice, *mdx* mice, as well as other models of DMD. Differences in reported forces can be due to a variety of both biological and experimental variations. Main biological variations include the mouse strain, age, and disease state, while prominent experimental variations include assessment of muscle dimensions and/or mass, frequency and/or duration of stimulation, sarcomere length, and experimental temperature. In an effort to allow straightforward quantitative comparison of distinct studies, TREAT-NMD (www.treat-nmd.eu) has developed an online resource detailing standard operating procedures (SOPs) for animal research within the neuromuscular field, including specific SOPs for work with *mdx* mouse diaphragm muscle. One of the many up-sides of a standardized protocol is that when such studies are ideally performed under identical conditions, results should be directly comparable between studies from different groups of investigators. In addition, a standardized protocol greatly aids in providing advice and background information especially for beginning scientists. A potential down-side is that creating a field-wide standard protocol for such tissue work could make it more difficult to pursue protocols that are different because of limitations or advancements in methods and technology, and do not strictly adhere to the guidelines. Thus, analogous to widely used guidelines for clinical best practices, regular revisiting of the standard procedures should be common practice for the field, and deviation of the protocol should not be a reason *per se* to downgrade the scientific impact of the experimental protocol. Here we present an argument for revising SOPs involving mouse diaphragm muscle to be preferably performed at 37°C.

The temperature at which muscle tissue is evaluated is an experimentally key parameter that often differs between a living organism and the laboratory setting. The majority of studies using mouse diaphragm tissue operate at or near room temperature, approximately 20–25°C (Stedman et al., 1991; Coirault et al., 1999a; Stevens and Faulkner, 2000; Gregorevic et al., 2002; Gosselin and Williams, 2006; Blaauw et al., 2009). Performing experiments at room temperature is done for many reasons. Historically, the vast majority of muscle physiology was performed on cold-blooded animals, mainly the frog. When in the latter part of the last century mammals became more widely-used model systems because of their greater resemblance with human physiology, the temperature of the experiments was often maintained at room temperature in order to compare to past work, as well as for experimental ease. Despite this advancement, assessment of contractile parameters at temperatures well below those that prevail *in vivo* (typically 36–39°C depending on species), is an obvious departure from the physiological prevailing setting. With the current increasing pressure on scientists to incorporate translational aspects into basic science, assessment of contractile performance at physiological rather than non-physiological temperature would allow for a more straightforward interpretation of *in vitro* experimental results to the *in vivo* situation and facilitate the development of possible enzymatic or pharmaceutical treatments that require higher temperatures.

Here, we sought to determine, under conditions close to described in the Treat-NMD SOP for *in vitro* contractile force assessment, how temperature affects the twitch and tetanic force as well as kinetics of the *mdx* mouse and wild-type comparison C57BL/10 mouse diaphragm, and therefore indirectly the ways in which it can affect study design. We found that the forces generated at body temperature by the wild-type and *mdx* diaphragm were slightly higher and developed drastically faster than at room temperature for both mouse strains. Also, as expected, the *mdx* diaphragm was significantly weaker compared to the wild-type at both temperatures. Because the relative difference between healthy controls and *mdx* mice was at least as great as at room temperature, achieving statistical significance does not require a greater number of animals.

## Materials and methods

### Mice

C57BL/10ScNHsd mice purchased from Harlan Laboratory and *mdx* (C57BL/10 ScSn DMDmdx) mice from The Jackson Laboratory were housed in the animal facilities of The Ohio State University College of Medicine with constant temperature and humidity and fed a standard diet. All protocols were approved by The Ohio State University Laboratory Animal Care and Use Committee. C57BL/10 mice were 9–11 weeks old at the time of sacrifice and *mdx* mice were 10 weeks old.

### Diaphragm force measurements

**Figure 1:**
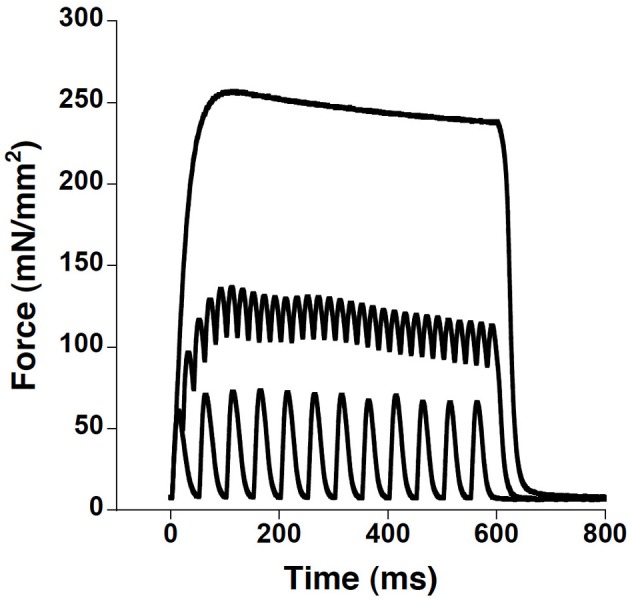
**Wild-type diaphragm strip contraction kinetics performed at 37°C at frequencies of 20, 50, and 180 Hz showed that at a frequency of 180 Hz, a smooth plateauing tetanus is achieved, in most preparations the smooth tetanus is already achieved at 120–150 Hz (not shown).** Since at decreasing temperature the tetanus frequency to plateau is lower, using 180 Hz tetani for all temperature ensured maximal activation of the preparation.

After sacrificing the mouse (cervical dislocation), the thorax was opened and the diaphragm and ribcage were removed and placed in a modified Krebs-Henseleit solution [95%O_2_/5%CO_2_ (pH 7.4), 5 mM KCl, 137 mM NaCl, 1.2 mM NaH_2_PO_4_, 1.2 mM MgSO_4_, 20 mM NaHCO_3_, 10 mM D-glucose, and 0.25 mM CaCl_2_] with the addition of 20 mM 2,3-butandione monoxime (BDM) to prevent muscle damage during dissection (Mulieri et al., 1989). Two linear strips of muscle, approximately 2–3 mm in width, were carefully dissected from each diaphragm. This width was found to be optimal through earlier unpublished work in our lab. On one end of the muscle the rib tissue was left intact, allowing the diaphragm strip to be held in place by inserting the muscle through a stainless steel basket connected to a force transducer (KG2, Scientific Instruments). The central tendon at the deep end of the diaphragm strip was then pierced over a hook that was connected to a linear micro-manipulator. Each muscle bath contained the same oxygenated Krebs-Henseleit solution as described above (without BDM and with 2.0 mM CaCl_2_) at either 25°C or 37°C. A single electrical stimulation pulse was delivered via two parallel platinum-iridium electrodes on either side of the muscle. The muscle was then stretched to its optimal length, defined as the length at which maximum twitch force is measured. The muscles were subsequently allowed to rest for 10 min. Diaphragm strips were run in one of two experimental set-ups: the temperature of each set-up was altered so that some experiments started at 25°C and were warmed to 37°C, while the other bath started at 37°C and was cooled to 25°C over the course of data collection. From each mouse one or two strips were dissected; if two were dissected, one was run in the warming bath and the other was run in the cooling bath. After equilibration and finding optimal length using twitch contractions, the muscles were subjected to a protocol consisting of a series of seven tetanic contractions with 180 Hz stimuli at maximal voltage for 250 ms occurring at intervals of 2°C, with approximately 3 min between contractions. The 180 Hz frequency and 250 ms duration were chosen from pilot studies that indicated this protocol-induced a smooth tetanus that reaches plateau at all temperatures (Figure 1). For comparative purposes, all force measurements are expressed per unit of cross-sectional area (CSA) (normalized isometric force or tension: mN/mm^2^). CSA was calculated using the following equation: muscle mass in mg/[(optimal length in mm) × (muscle density in mg/mm^3^)], where muscle density is assumed to be 1.056 mg/mm^3^.

In a separate cohort of animals (*n* = 11 C57BL/10 and *n* = 8 *mdx* mice), we assess a response to a fatigue/injury protocol at 37°C. Muscles were stimulated with 500 ms tetani followed by 500 ms or rest every second for 1 min. Thereafter, the muscles were left to rest unstimulated for 15 min, and the 1 min on-off protocol was repeated.

### Data analysis

Differences in strip parameters and single-temperature comparisons were evaluated via *t*-tests using Microsoft Excel. Two-factor repeated measures ANOVA tests were run using SPSS (SPSS, Inc., Chicago, IL) to compare the warming vs. cooling trend within each strain, the force and timing comparison between *mdx* and C57BL/10 groups over the range of temperatures.

## Results

**Figure 2:**
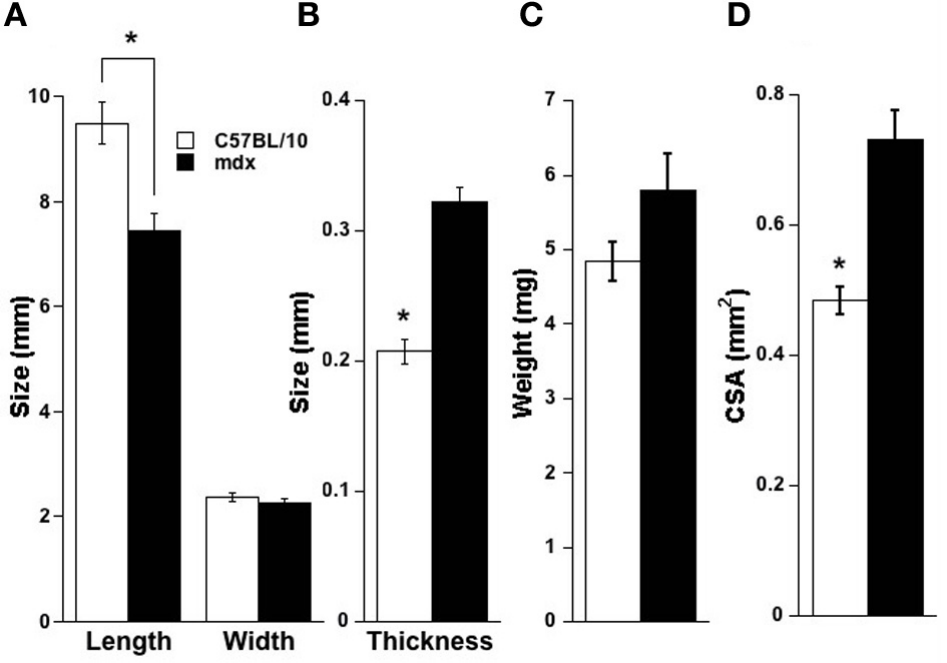
**Diaphragm strip parameters. (A)** C57BL/10 strips were stretched to longer lengths (*p* < 0.01), owing to lower elasticity in the *mdx* diaphragm (Stevens and Faulkner, 2000) compared to C57BL/10, and width did not vary (*p* = 0.43). **(B)** The calculated thickness of strips was higher for *mdx* tissue vs. C57BL/10 (*p* < 0.01). **(C)** Strip mass was not statistically different between C57BL/10 and *mdx* (*p* = 0.07) but cross-sectional area based on mass of the strip (mass divided by the product of muscle density and length) was higher for *mdx* diaphragm strips vs. C57BL/10 (*p* < 0.01), shown in panel **(D)**. ^*^ indicates *p*<0.05 between *mdx* and C57BL/10.

Data were collected from 16 C57BL/10 mice and 10 *mdx* mice with two muscle strips taken from each diaphragm. From the 10 C57BL/10 mice, 6 strips were run from 37°C to 25°C (cooling) and 10 from 25°C to 37°C (warming). From the 10 *mdx* strips there were 5 strips run in each direction. Average muscle strip parameters are shown in Figure 2. Strips from both mouse strains were cut to nearly identical widths (2.4 vs. 2.3 mm for C57BL/10 and *mdx*, respectively, Figure 1). C57BL/10 mice had a longer average diaphragm strip optimal length (*p* < 0.01, Figure 2A). The average *mdx* CSA is significantly higher compared to C57BL/10 (*p* < 0.01, Figure 2D) and the difference in mass approaches statistical significance (*p* = 0.07, Figure 2C). Also, shown in Figure 2B is the average calculated strip thickness. This parameter is calculated as CSA (determined as mass divided by the product of muscle density and length) divided by width. This calculation provides an estimated strip thickness assuming the cross-section is rectangular. C57BL/10 mice have a significantly smaller calculated strip thickness than *mdx* mice (*p* < 0.01). Strip thickness was not measured directly.

**Figure 3:**
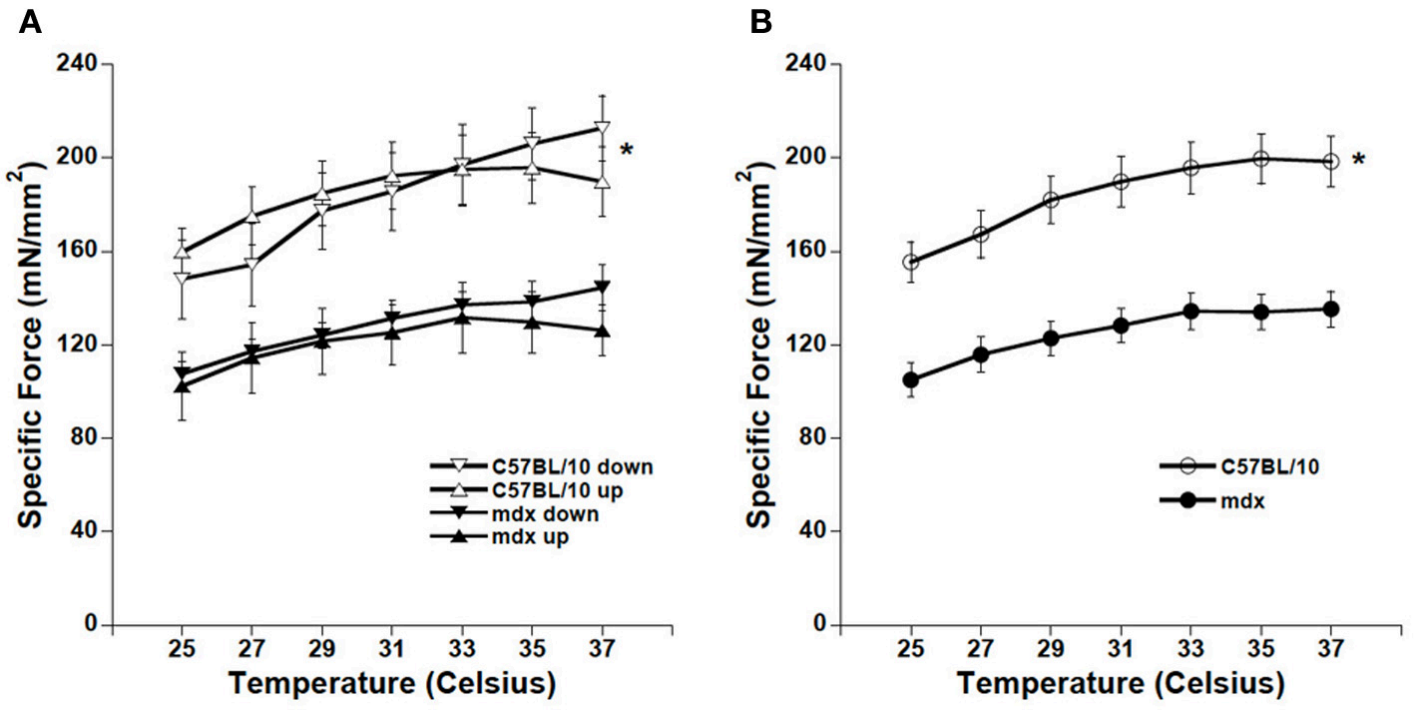
**(A)** Warming/cooling force comparisons for C57BL/10 and *mdx* diaphragm strips. Force increased significantly with temperature for the C57BL/10 group (*p* = 0.01) but not quite significantly in the *mdx* group (*p* = 0.06). Neither C57BL/10 (*p* = 0.84) nor *mdx* (*p* = 0.24) showed differences in force when comparing within-strain warming vs. cooling groups. **(B)** Pooled data shows that C57BL/10 diaphragm strips showed higher average force per CSA vs. *mdx* (^*^p < 0.01) at all temperatures, and a significant relationship between temperature and force (*p* < 0.01).

The force-temperature relationship is shown for the C57BL/10 and *mdx* diaphragm in Figure 3. Both data sets show a trend toward increasing force per CSA with an increase in temperature (*p* = 0.01 for C57BL/10 and *p* = 0.06 for *mdx*). There is no difference in force comparing warming vs. cooling in the C57BL/10 group (*p* = 0.84) nor is there a difference within the *mdx* group (*p* = 0.24). Thus, warming and cooling data from the same strain were grouped together for comparison (Figure 3B). From the pooled data we derive that the overall force/CSA is higher among the C57BL/10 mice compared to *mdx* (ANOVA, *p* < 0.01) independent of temperature, and there is a significant trend toward increasing force with increasing temperature (ANOVA, *p* < 0.01) for the overall data set.

**Figure 4:**
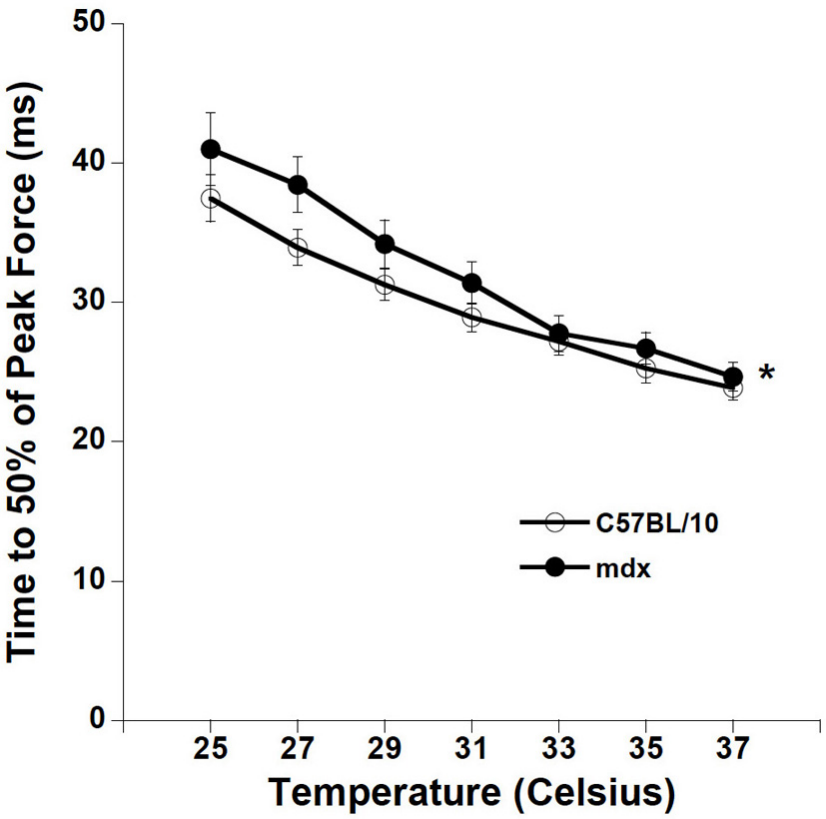
**Temperature dependency of C57BL/10 and *mdx* tetanic contraction kinetics.** Time from first stimulation to 50% of maximal force is quicker with increasing temperature for tetanic contractions in all groups (*p* < 0.01 for TT50).

We proceeded to quantify the contraction kinetics of the tetani. For each tetanic contraction we calculated the time from start of first stimulation to 50% of peak force for contractions at optimal length. *mdx* strips had slower T50 compared to C57BL/10 diaphragm muscle strips (ANOVA, *p* < 0.05), as depicted in Figure 4.

**Figure 5:**
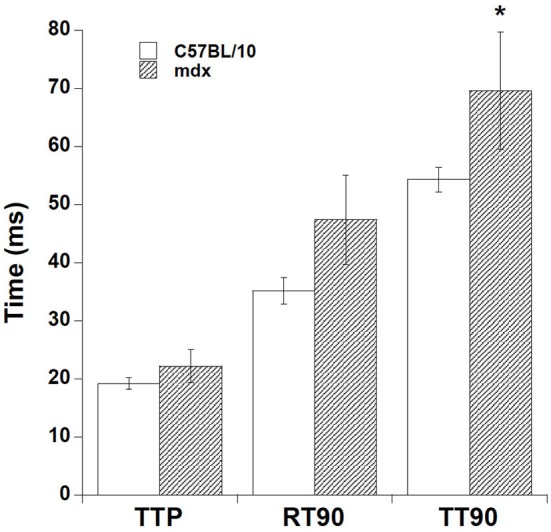
**Twitch-kinetic properties at 37°C.** Time to peak tension (TTP) was slightly less in C57BL/10 strips vs. *mdx* strips, while time from peak tension to 90% relaxation (RT90) showed a similar trend. As a result, overall duration of contraction (TT90, taken from stimulation to 90% relaxation), was significantly longer in *mdx* vs. C57BL/10 muscle strips ^*^(*p* < 0.05, 69.6 ± 10.1 vs. 54.3 ± 2.1 ms).

In addition to the tetanic contraction, we investigated the twitch-kinetic properties at 37°C (Figure 5). At optimal length, time to peak tension (TTP) was slightly faster in C57BL/10 strips vs. *mdx* strips, while time from peak tension to 90% relaxation (RT90) showed a similar trend. As a result, overall duration of contraction (TT90, taken from stimulation to 90% relaxation), was significantly longer in *mdx* vs. C57BL/10 muscle strips (54.3 ± 2.1 vs. 69.6 ± 10.1 ms, *p* < 0.05) at 37°C. From a subset of experiments where tetanic and twitch forces were assessed at both 25 and at 37°C, we calculated the Q10's to be (on average, no significant difference between *mdx* and C57BL/10): Tetanic force development 1.07, twitch force development 1.22, and twitch kinetics 2.86.

**Figure 6:**
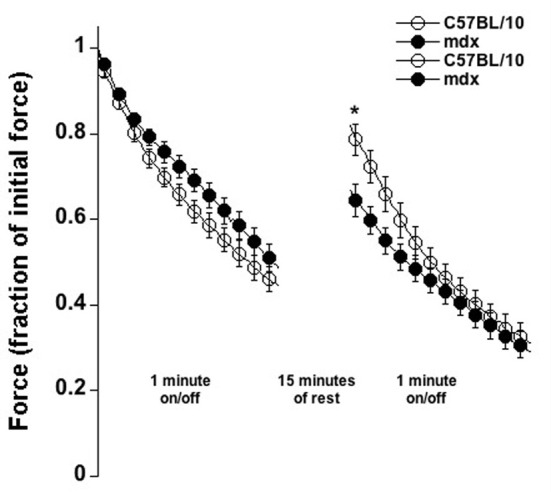
**Fatigue/injury-induced by 60 repetitive contractions (each 500 ms tetanus followed by 500 ms rest), followed by 15 min of rest and a repeat in C57BL/01 and *mdx* muscles at 37°C.** Overall fatigue is similar in both groups, but the recovery of force is 2-fold better in C57BL/10 muscles compared to *mdx* muscles (*P* < 0.01).

Last, we tested a fatigue/injury protocol in which muscles were stimulated 500 ms followed by 500 ms rest for 60 consecutive seconds (Figure 6). The relative force-drop due to fatigue/injury was the same between C5Bl/10 and *mdx* mice. After 15 min of rest however, the C57BL/10 muscles had significantly recovered more by an average of 37.4% of initial force vs. only 18.1% in *mdx* muscles (*p* < 0.01), indicating that either the recovery in *mdx* muscle is significantly slower, or that a larger permanent amount of injury was inflicted in *mdx* muscles by this protocol, or potentially by a combination of these two factors.

## Discussion

We show here that the difference in the contractile profile between wild-type mice and *mdx* mice is virtually identical at either 25°C or 37°C. However, a significant number of potential treatments, such as enzymes and drugs, do not necessarily behave quantitatively or qualitatively similar at both temperatures: there are many biological processes that are significantly impaired at low temperature. Thus, despite the vast similarities in quantitative outcome in non-treated control and *mdx* diaphragm strips, using experiments performed at physiological temperature may potentially provide a less ambiguous extrapolation of *in vitro* contractile data to the *in vivo* situation.

The collecting of contractile data from diaphragm strips at body temperature has not been standardized for several reasons. For one, *in vitro* experiments performed at 37°C have been labeled unstable or variable in the past. We here demonstrate that reproducible, stable, and quality data can effectively be obtained. In our hands, it is indeed true that at 37°C a mechanical injury (e.g., dissection injury) leads to a more substantial injury than at much lower temperature. This is somewhat analogous to past experience by us and others with *in vitro* cardiac muscle preparations when a cardiac muscle cell is injured at 37°C, the rapid spread of injury due to mechanical stress and calcium overload on neighboring cells causes an overall injury pattern that is more severe than when a comparable injury happens at low temperature, where spreading of injury is slower (Backx and Ter Keurs, 1993; Janssen et al., 2002; Raman et al., 2006). However, this physiological temperature does not make the preparation unstable *per se*, it simply fails to mask a dissection/handling injury well. Although it could be labeled “unstable,” one could argue that the advantage of experiments at 37°C is that they allow for a better judgment of preparation injury. Although using different protocols, other laboratories too are also using 37°C for contractile assessment of diaphragm strips, indicating that successful experimentation under more physiological conditions is reliable and reproducible (Prezant et al., 1990; Diaz et al., 1994; Brooks et al., 2002; Rucker et al., 2004). From our data in this study, we analyzed the decrease in force during the experiments, averaging an equal number of experiments starting at 25°C and at 37°C, and were able to calculate that over the course of seven tetani, each 2–3 min apart loss of force was <1.5% per contraction (<0.8% for each of the first 4 tetani, ~2.2% for each of the last 3), indicating stable contractile parameters of such *in vitro* preparations, even at 37°C.

A second reason that preparations are often used only at cold temperature is the argument that the results can now be compared with data obtained in the past. Clearly, this is an argument that can play a decision in design of a protocol for a new set of experiments, but if this is the only argument used, it hampers progress of the field. In addition, an increasing focus on clinical translational science has prompted an increase in the number of recent studies being performed under physiologically relevant temperature (Chandrasekharan et al., 2010; Lawler et al., 2010; Beastrom et al., 2011; Peterson et al., 2011; Rafael-Fortney et al., 2011), as it reflects the *in vivo* temperature at which the diaphragm operates. We thus predict that the increased importance of translational science will result in an expanding data base to which new data can and should be compared. Thus, we would suggest that only when there are compelling reasons other than the comparison with past cold-temperature work, studies on isolated diaphragm contractile function should be executed at a physiologically relevant temperature.

In this study, the diaphragm strips from *mdx* mice had a higher average mass and thickness and a shorter optimal length as compared to C57BL/10 controls. It is indeed well known that *mdx* muscle tissue is hypertrophied (Gillis, 1999), an observation also found in human DMD patients (Emery, 2002). Additionally, it has been shown previously that diaphragm tissue from *mdx* mice has less elasticity and can undergo less stretch than C57BL/10 tissue (Stevens and Faulkner, 2000). It is not surprising then to observe comparatively short, thick strips from the *mdx* diaphragm vs. those isolated from the C57BL/10 mouse. It has also been previously reported that the *mdx* diaphragm produces less force per CSA than wild-type, and this reduction in CSA-based force is a phenotypical hallmark of this disease (Stedman et al., 1991; Cox et al., 1993; Stevens and Faulkner, 2000; Harcourt et al., 2007; Peterson et al., 2011). In the present study, we observed that at the physiological core temperature of the mouse (37°C), both *mdx* and C57BL/10 diaphragm produced slightly more tetanic force per CSA than at 25°C. Previous studies on tetanic force too have shown only little, but no significant, difference between room temperature and body temperature (Prezant et al., 1990; Diaz et al., 1994; Brooks et al., 2002). Here, using a paired-testing approach, where each muscle was studied at both 25°C and 37°C, we find the difference to be (borderline) statistically significant. The difference between *mdx* and WT strips was virtually identical, at 25°C, C57BL/10 strips produced on average 45.9% more tetanic force per CSA than *mdx* strips, while at 37°C this average difference was 45.1%. The obtained forces in this study (~200 mN/mm^2^ for healthy strips) are very similar to those reported by others, and the difference between C57BL/10 and *mdx* strips is in the expected range and in agreement with previously published values comparing *mdx* and wild type of 47% (Peterson et al., 2011), 49% (Petrof et al., 1993b), 31% (Beastrom et al., 2011), 66% (Harcourt et al., 2007), 50% (Coirault et al., 1999b), 70% (Deconinck et al., 1998), and 54% (Coirault et al., 2003) (various protocols and ages). Thus, both in level of tetanic force development, as well as in the difference between control and *mdx* diaphragm strips, results are very similar to those previous studies at predominantly non-physiological temperatures.

It should be noted that factors other than temperature affect quantitative outcome of diaphragm muscle strip contractions. It is unavoidable that fibers on the edges of the strips get damaged during dissection, thus the larger the preparation, the smaller the proportion of edges. However, because larger preparations can impede the flow of solution in our muscle bath, they are harder to sufficiently oxygenize via diffusion, and cannot get rid of waste products as easily. As a result, preparations that are too large can suffer from core-hypoxia or contractile inactivation by waste products, similar to as has been shown for *in vitro* cardiac muscle preparations (Schouten and ter Keurs, 1986; Raman et al., 2006). In our hands, preparations of between 2 and 3 mm in width provided the highest amount of specific force, dimensions that are in close agreement with the suggested SOP. Lastly, especially when a preparation is needed for histological analysis a forced blotting of the preparation is often avoided. As a consequence, if mass rather than dimension is used for determination of cross-sectional area, any amount of solution attached to the preparation can cause overestimation of mass, and thus underestimation of specific force. Conversely, an overly strong blotting of the preparation may cause underestimation of true normal tissue mass, and result in overestimation of specific force. Specific forces can be reliably calculated in diaphragm strips when length, thickness and mass are assessed.

It is well known that temperature significantly increase the kinetics of striated muscle contraction, including in the diaphragm (Diaz et al., 1994). Both the kinetics from the twitch contractions as well as those of the tetanic contractions are about 2–3 times as fast at 37°C in both groups when compared to 25°C. In addition to the rise-time of the tetanic contraction, at 37°C the twitch contraction is prolonged in *mdx* muscles compared to wild-type muscles, in line with previous studies executed below physiological temperature (Deconinck et al., 1998; Gregorevic et al., 2002; Coirault et al., 2003; Harcourt et al., 2007). Due to the faster kinetics, at 37°C a 180 Hz tetanus reaches maximal plateau force already at ~80–120 ms. Thus, the 250 ms (or often longer) tetanus protocol that is typically needed to reach plateau force at 25°C and used in this study could be further abbreviated to only 100–150 ms in future studies at 37°C. The significantly faster kinetics have the advantage that fatigue or run-down of the preparation can potentially be further minimized by applying shorter tetanic contractions to assess maximal force-generating capacity. Our fatigue injury protocol revealed that fatigue seemed to impact C57BL/10 muscles similarly to *mdx* muscles, whereas a 15 min rest and repeat of the protocol revealed that *mdx* muscles did either not recover as fast, or had sustained permanent injury. Pilot experiments with longer periods of rest (30 min) did not reveal further recovery of *mdx* nor C57BL/10 muscles. Thus, the fatigue/injury protocol, performed at 37°C, was able to distinguish the phenotypical difference between *mdx* and C57BL/10 mice, and indicated larger amounts of contraction-induced damage in *mdx* mice.

We conclude that *in vitro* contractions of diaphragm muscle strips form *mdx* and wild-type mice can be reliably and reproducibly performed at body temperature. We recommend altering existing SOPs to state experiments be preferentially performed at physiologically relevant temperatures wherever possible, which could facilitate translation of results to clinically relevant situations.

### Conflict of interest statement

The authors declare that the research was conducted in the absence of any commercial or financial relationships that could be construed as a potential conflict of interest.
